# Distinct Role of Mono-2-ethylhexyl Phthalate in Neuronal Transmission in Rat CA3 Hippocampal Neurons: Involvement of Ion Channels

**DOI:** 10.3390/molecules27103082

**Published:** 2022-05-11

**Authors:** Yi Lu, Hong Wang, Junqing Yang, Wengao Jiang, Hong Xin, Ying Luo, Santiago Loya-López, Huaiyu Gu, Dongzhi Ran

**Affiliations:** 1 Chongqing Key Laboratory of Biochemistry and Molecular Pharmacology, Department of Pharmacology, School of Pharmacy, Chongqing Medical University, Chongqing 400016, China; bubble2022@126.com (Y.L.); 101832@cqmu.edu.cn (H.W.); cqyangjq@cqmu.edu.cn (J.Y.); jiangwengao@cqmu.edu.cn (W.J.); 100894@cqmu.edu.cn (Y.L.); 2 Department of Pharmacy, People’s Hospital of Xinjin District, Chengdu 611430, China; anninglicc@163.com; 3 Department of Pharmacology, College of Medicine, The University of Arizona, Tucson, AZ 85724, USA; zzz20220322@163.com; 4 Department of Human Anatomy, Histology and Embryology, Peking Union Medical College, Beijing 100730, China; gu_huaiyu@yahoo.com

**Keywords:** Mono-2-ethylhexyl phthalate, neuronal transmission, hippocampal neurons, ion channels, neural excitability, synaptic plasticity

## Abstract

Mono-(2-ethylhexyl) phthalate (MEHP) is one of the main active metabolites of di-(2-ethylhexyl) phthalate (DEHP). In our previous works, by using rat and Drosophila models, we showed a disruption of neural function due to DEHP. However, the exact neural effects of MEHP are still unclear. To explore the effects of MEHP on the central nervous system, the electrophysiological properties of spontaneous action potential (sAP), mini-excitatory postsynaptic currents (mEPSCs), ion channels, including Na^+^, Ca^2+^, and K^+^ channels from rat CA3 hippocampal neurons area were assessed. Our data showed that MEHP (at the concentrations of 100 or 300 μM) decreased the amplitude of sAP and the frequency of mEPSCs. Additionally, MEHP (100 or 300 μM) significantly reduced the peak current density of Ca^2+^ channels, whereas only the concentration of 300 μM decreased the peak current density of Na^+^ and K^+^ channels. Therefore, our results indicate that exposure to MEHP could affect the neuronal excitability and synaptic plasticity of rat CA3 hippocampal neurons by inhibiting ion channels’ activity, implying the distinct role of MEHP in neural transmission.

## 1. Introduction

Mono-(2-ethylhexyl) phthalate (MEHP), one of the main primary metabolites of di-(2-ethylhexyl) phthalate (DEHP), has been detected at significant levels in urine [1]. Meanwhile, Cobellis’s study reported a higher concentration of MEHP in women with endometriosis compared with a control group [2]. In 2006, the FDA’s Center for Devices and Radiological Health (CDRH) reported that neonates have the risk of exposure to high doses of MEHP, due to blood transfusion or other life-saving procedures, because the medical devices may contain a high level of DEHP. DEHP, the most widely used plasticizer, is added in polyvinyl chloride (PVC) to render products softer and more malleable [3]. DEHP can be found in medical devices [4], food wrapping [5], cosmetics [3], and numerous other commercial products. However, as a small molecule compared to other large polymer chains, DEHP is not chemically bound to polymer chains [1], and can be quickly hydrolyzed into MEHP by esterase in the intestinal tract, and also in the liver, kidney, lungs, pancreas, and plasma [6]. Researchers have revealed that oral administration of DEHP could result in an MEHP concentration several orders of magnitude higher than that of the general population exposure level [7]. More importantly, as environmental-endocrine disrupting chemicals (EDCs), MEHP and DEHP consistently produce developmental, reproductive, immune, respiratory, and hepatic toxicity [8,9,10,11]. Moreover, MEHP is reported to be the ultimate toxicant of DEHP, and both DEHP and MEHP can induce apoptosis in the cells of immune system. However, the metabolite MEHP was found to be more toxic to TK6 lymphoblast cells than the parent compound DEHP [12]. Therefore, the effects of MEHP on human health cannot be ignored.

In addition to the effects of MEHP/DEHP on the organs and systems above, a strong association between MEHP/DEHP exposure and neurological impairment has begun to emerge. Our previous study showed that the DEHP could inhibit the cholinergic synaptic transmission of projection neurons (PNs) in Drosophila antennal lobes [13], and neuronal excitability and synaptic plasticity of rats’ hippocampal neurons [14], leading to spatial learning and memory impairment. Meanwhile, decreased expression of BDNF mRNA due to DEHP exposure might also lead to the reduction of dendritic spine density in hippocampal CA3 neurons [15], and prenatal exposure to DEHP has a gender-specific negative impact on the dendritic growth of CA1 pyramidal neurons in male offspring, suggesting the effects of DEHP on the hippocampus. Moreover, after DEHP or DEHP metabolite exposure, adult rats showed significant disruptions in ion homeostasis by the inhibition of membrane Na^+^/K^+^-ATPase activity, leading to neuronal degeneration [16], indicating the ion channel induced regulation of hippocampal function by DEHP or DEHP metabolites. As one of the main active metabolites and ultimate toxicants of DEHP, MEHP may have the potential to impede the nervous system’s function. Nevertheless, the exact neurological effects of MEHP are still unclear. Thus, digging out the effects of MEHP on the nervous system and investigating the mechanisms involved is essential for human health.

The hippocampus is a major component of the brain of humans and other mammals [17]. In particular, the axons of pyramidal cells in the CA3 region of the hippocampus spread within most of the region to form an associative neuronal network [18]. Normally, because pyramidal cells in the CA3 region excite other pyramidal cells and interneurons [19], signals from dentate gyrus are sent to the CA3 subregion, and then projected to the CA1 region [20]. Therefore, the key features of CA3 pyramidal cell might be important to the neural function. Given these studies, a consideration of the pivotal role of MEHP in the CA3 region is of interest. Therefore, in the present study, the impacts of MEHP on the neural excitability and synaptic plasticity of CA3 pyramidal cells and possible mechanisms involved have been detected.

In our previous studies [13,14,21], in order to make sure that we can observe as many toxic effects of DEHP or MEHP on neurons as possible, the higher concentrations (100, 300 μM) of exposure after an exchange transfusion procedure [22] were chosen. According to our research published in 2021 [21], at a dose of 300 μM, but not 100 μM, MEHP was showed to inhibit the excitability of projection neurons in Drosophila antennal lobes, and reduce the peak current densities of sodium and calcium channels. Therefore, the same doses (100, 300 μM) of MEHP were chosen to continue to explore its effects on the electrophysiological properties of neural transmission of rats’ hippocampal CA3 neurons. Through the MEHP treatments, spontaneous action potential (sAP), representing the ability of neural excitability and neural transmission, ion channels, including voltage-gated sodium ion channel (Nav), voltage-gated potassium ion channel (Kv), and voltage-gated calcium ion channel (Cav), which are the elementary factors of APs, as well as mini-excitatory postsynaptic current (mEPSC), which is the electrophysiological measurement of synaptic plasticity, reflecting the functional and/or structural aspects of a synapse [23], have been recorded and analyzed to investigate the role of such a compound in the neural circuit transmission and elucidate the electrophysiological mechanism involved.

## 2. Results

### 2.1. MEHP Suppressed the Excitability of Rat Hippocampal CA3 Neurons

**Figure 1:**
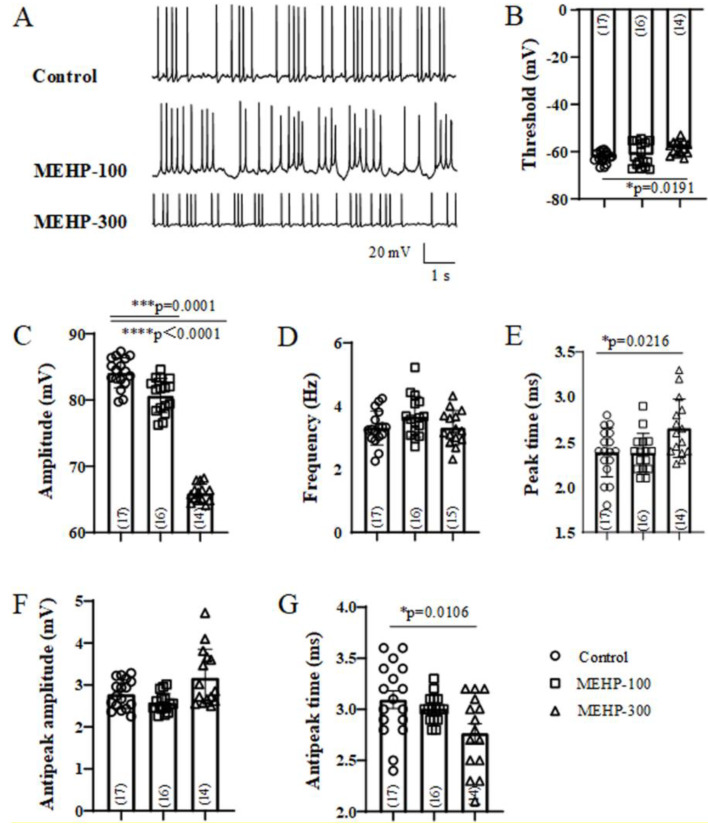
Effects of MEHP at different concentrations on sAP recorded from rat CA3 hippocampal neurons. In current-clamp mode, sAP was recorded with a holding potential at −70 mV. sAP from control (n = 17), MEHP-100 (n = 16), and MEHP-300 (n = 14) groups were recorded. Representative sAP traces are shown (**A**). sAP properties of CA3 neurons, in terms of the potential threshold necessary for sAP generation (**B**), amplitude (**C**), frequency (**D**), peak time (**E**), antipeak amplitude (**F**), and antipeak time (**G**), have been detected. sAP was recorded with a standard external solution containing (in mM): 140 NaCl, 1.5 CaCl_2_, 5 KCl, 1 MgCl_2_, 10 HEPES, and 10 glucoses (pH 7.2~7.4). Pipette electrodes were filled with internal solution containing (in mM): 140 potassium gluconate, 5 NaCl, 2 MgATP2, 1 CaCl_2_, 10 EGTA, and 10 HEPES (pH 7.2–7.4, 310 mOsm). The data were expressed as the mean ± SEM. Each bar indicates the mean ± SEM from the indicated number of neurons. (* means *p* < 0.05, *** means *p* < 0.001, and **** means *p* < 0.0001).

AP plays an essential role in the transmission of neurons and maturation of neural circuits [24]. To investigate the effects of MEHP on the excitability and formation of neural circuits, we recorded the sAP from rat hippocampal CA3 neurons (Figure 1). The representative traces of sAP from control, MEHP-100 and MEHP-300 groups are showed in Figure 1A. The potential threshold was decreased significantly to −57.43 ± 0.76 mV by the application of MEHP-300 (n = 14, *p* < 0.05), but not MEHP-100 (n = 16, *p* > 0.05) (Figure 1B). Moreover, the amplitudes of sAP have been significantly decreased to 80.67 ± 0.65 mV (MEHP-100, n = 16, *p* = 0.0001) and 65.95 ± 0.36 mV (MEHP-300, n = 14, *p* < 0.0001) (Figure 1C) compared with the control group. With the application of MEHP-100, none of the sAP frequency (n = 16, *p* > 0.05, Figure 1D), peak time (n = 16, *p* > 0.05, Figure 1E), antipeak amplitude (n = 16, *p* > 0.05, Figure 1F), or antipeak time (n = 16, *p* > 0.05, Figure 1G) differed from those of the control. However, with the MEHP-300 treatment, compared with the control group, sAP peak time increased to 2.65 ± 0.08 ms (n = 14, *p* < 0.05, Figure 1E), and antipeak time decreased to 2.77 ± 0.09 ms (n = 14, *p* < 0.05, Figure 1G), whereas the frequency (n = 14, *p* > 0.05, Figure 1D) and antipeak amplitude (n = 14, *p* > 0.05, Figure 1F) did not change. Together, these data showed that MEHP altered the properties of sAP in CA3 neurons. Moreover, it also implied that MEHP may have the potential to influence the ion channels, including the sodium channel, potassium channel, and calcium channel, which participate in the formation of sAP.

### 2.2. MEHP Decreased the Na^+^ Current Recorded from Rat CA3 Hippocampal Neurons

**Figure 2:**
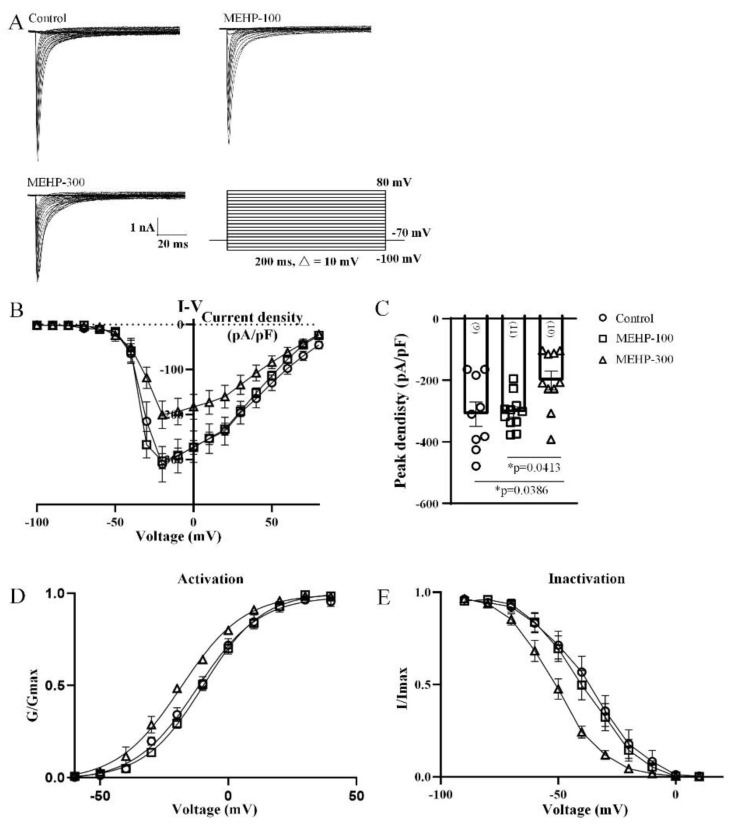
Effect of MEHP at different concentrations on the sodium channel activities recorded from rat CA3 hippocampal neurons. (**A**) shows the representative traces of the sodium channel activities of control, MEHP-100, and MEHP-300 groups. (**B**,**C**) show the average current densities and peak current densities of neurons from control (n = 6), MEHP-100 (n = 11), and MEHP-300 (n = 10) groups, respectively. (**D**,**E**) show the activation curves and inactivation curves of the sodium channel recorded from neurons of the control, MEHP-100, and MEHP-300 groups, respectively. Na+ currents were recorded with an external solution containing (in mM): 140 NaCl, 1 MgCl_2_, 5.0 KCl, 3.0 CaCl_2_, 10 TEA, 1 4-AP, 0.2 CdCl_2_, 10 HEPES, 10 glucose with pH adjusted to 7.2~7.4 using NaOH (mOsm 290~300). Pipette electrodes were filled with internal solution containing (in mM): 140 CsCl, 10 TEA, 10 EGTA, 10 HEPES with pH adjusted to 7.2~7.4 using CsOH. The data are expressed as the mean ± SEM. Each bar indicates the mean ± SEM from the indicated number of neurons. (* means *p* < 0.05).

The sodium channel matters greatly in the formation and maintaining of sAP, especially the membrane potential threshold, amplitude, and peak time of sAP [25]. Meanwhile, Figure 1 showed that MEHP might have the possibility to influence the properties of sAP. Therefore, to further verify whether MEHP has the potential to affect the ion channels, we detected the sodium channel recordings from CA3 hippocampal neurons and analyzed the average current densities, peak current densities, activation, and inactivation curves of the sodium current (Figure 2). In a voltage-clamp protocol, CA3 neurons were held at −70 mV with depolarization steps (200 ms step) from −100 mV to +80 mV in 10 mV increments (Figure 2A). The representative traces in Figure 2A showed a family of sodium currents recorded from CA3 neurons in the control, MEHP-100, and MEHP-300 groups (Figure 2A). Compared with the control group, MEHP-300, but not MEHP-100 reduced the average sodium currents and the peak currents density (Figure 2B,C). To determine whether changes in channel gating are the causes of the inhibitory properties of MEHP on Na^+^ currents, we detected the effects of MEHP on the biophysical properties of activation and inactivation of sodium currents. With the treatments of MEHP-300, the sodium channel activation and inactivation curve both shifted to the left, indicating the sodium channels opened earlier and closed later, compared with the control group (Figure 2D,E). In conclusion, these data showed that MEHP could decrease sodium channel activity of CA3 neurons.

### 2.3. MEHP Decreased the Total Kv Current Recorded from Rat CA3 Hippocampal Neurons

**Figure 3:**
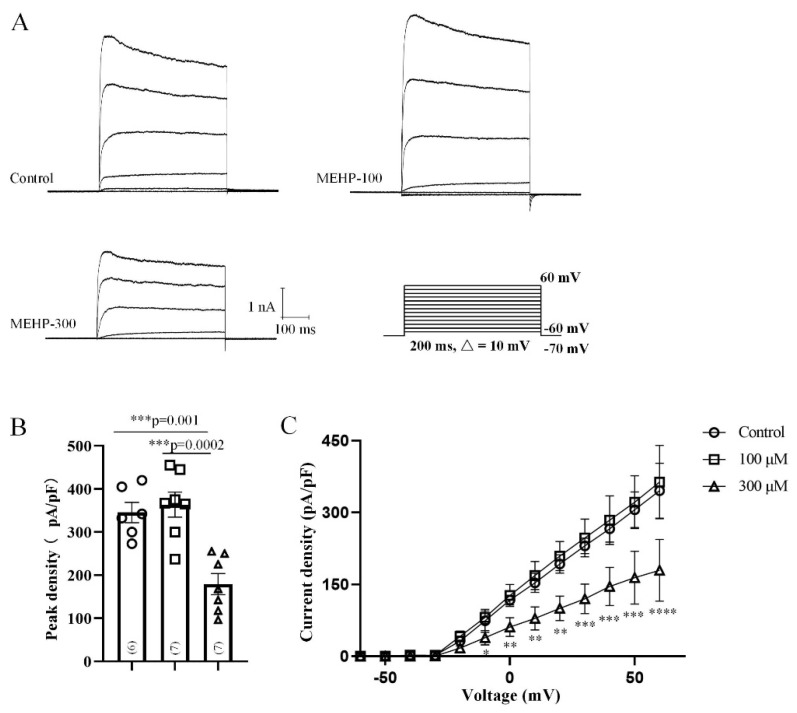
Effect of MEHP at different concentrations on the total Kv currents recorded from rat CA3 hippocampal neurons. (**A**) shows the representative traces of the total Kv currents of the control, MEHP-100, and MEHP-300 groups. (**B**) shows the peak current densities of neurons from the control (n = 6), MEHP-100 (n = 7), and MEHP-300 (n = 7) groups, respectively. (**C**) shows the current-voltage curve of total Kv currents in the control MEHP-100 and MEHP-300 groups. Kv currents were recorded with an external solution containing (in mM): 140 NaCl, 1.0 MgCl_2_, 5.0 KCl, 3 CaCl_2_, 0.001 TTX, 10 HEPES, 10 glucose with pH adjusted to 7.2~7.4 using NaOH (mOsm 300~310). Pipette electrodes were filled with internal solution containing (in mM): 140 KF, 10 EGTA, 10 HEPES with pH adjusted to 7.2–7.4 using KOH (mOsm 290~300). The data are expressed as the mean ± SEM. Each bar indicates the mean ± SEM from the indicated number of neurons. (*** means *p* < 0.001).

The potassium channel is a key factor of APs’ width and threshold [26]. Our sAP data showed that the threshold and peak time have been changes with the MEHP-300 treatment, indicating MEHP could regulate the potassium channel. To investigate the influences of MEHP on the potassium channel, we recorded the total Kv currents from CA3 neurons (Figure 3). By using the voltage-clamp protocol, we held CA3 neurons at −70 mV with depolarization steps (200 ms step) from −60 mV to +60 mV in 10-mV increments (Figure 3A). The representative traces of CA3 neurons in the control, MEHP-100, and MEHP-300 groups were shown in Figure 3A. The average potassium currents’ density and peak potassium currents’ density have been significantly influenced by MEHP-300, but the activation and inactivation were not affected by the treatment of either MEHP-100 or MEHP-300 (data not shown). Therefore, these data indicated the possibility that MHEP could affect the formation and maintaining of sAP by regulating Kv channels.

### 2.4. MEHP Decreased the Total Calcium Current Recorded from Rat CA3 Hippocampal Neurons

**Figure 4:**
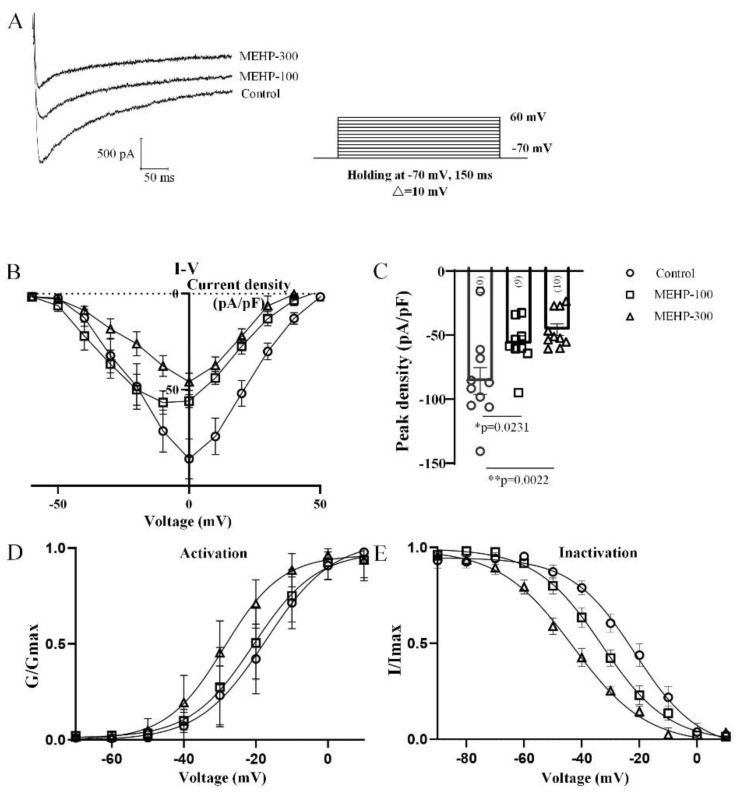
Effect of MEHP at different concentrations on the calcium channel activities recorded from rat CA3 hippocampal neurons. (**A**) shows the representative traces of the calcium channel activities of control, MEHP-100 and MEHP-300 groups. (**B**,**C**) show the average current densities and peak current densities of neurons from the control (n = 10), MEHP-100 (n = 9), and MEHP-300 (n = 10) groups, respectively. (**D**,**E**) show the activation curves and inactivation curves of the calcium channel recorded from neurons of the control group, MEHP-100, and MEHP-300 groups, respectively. Ca^2+^ currents were recorded with an external solution containing (in mM): 140 NaCl, 1.0 MgCl_2_, 5.0 KCl, 3 CaCl_2_, 10 TEA, 1 4-AP, 0.001 TTX, 10 HEPES, 10 glucose with pH adjusted to 7.2~7.4 using NaOH (mOsm 300~310). Pipette electrodes were routinely filled with internal solution con-taining (in mM): 140 CsCl, 10 TEA, 2 Na2ATP, 10 EGTA, 10 HEPES with pH adjusted to 7.2~7.4 using CsOH (mOsm 290~300). The data are expressed as the mean ± SEM. Each bar indicates the mean ± SEM from the indicated number of neurons. (* means *p* < 0.05, ** means *p* < 0.01).

The calcium channel is known to be associated with the posthyperpolarization of APs [27]. After MEHP-300 exposure, the antipeak time of sAP has been significantly decreased compared to the control group (Figure 1), implying that MEHP might affect the calcium channel. Thus, to investigate the effects of MEHP on calcium channels in CA3 neurons, we analyzed the average current densities, peak current densities, and activation and inactivation curves of the calcium current from CA3 neurons (Figure 4). Based on the voltage-clamp protocol, we held CA3 neurons at −70 mV with depolarization steps (150 ms step) from −70 mV to +60 mV in 10-mV increments (Figure 4A). The representative traces shown in Figure 4A were recorded from the CA3 neurons in the control, MEHP-100, and MEHP-300 groups. Compared with the control group, the average calcium currents’ density has been significantly inhibited with MEHP-100 and MEHP-300, and the peak currents’ density has been decreased significantly (MEHP-100, n = 9, *p* < 0.05, MEHP-300, n = 10, *p* < 0.05, Figure 4B,C). Furthermore, MEHP also has effects on the biophysical properties of activation and inactivation of calcium currents. The calcium channel activation and inactivation curve both shifted to the left, as a result of the MEHP-100 and MEHP-300 treatments, indicating the potential effects of MEHP on the potassium-activated calcium channel (Figure 4D,E). Summarily, these results showed that MEHP could influence calcium channel activity in CA3 neurons, including the inhibition of Ca^2+^ currents and the changes in calcium channel gating.

### 2.5. MEHP Reduced the Frequency of mEPSC Recorded from Rrat CA3 Hippocampal Neurons

**Figure 5:**
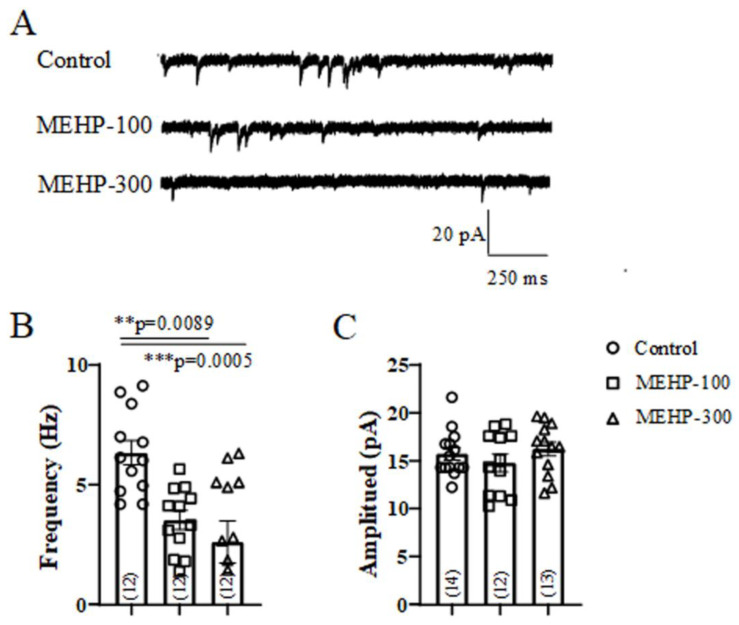
Effect of MEHP at different concentrations on the mEPSC amplitude and frequency recorded from CA3 pyramidal cells rat CA3 hippocampal neurons. (**A**) shows the representative traces of the mEPSC from the control, MEHP-100, and MEHP-300 groups. (**B**,**C**) show the amplitude and frequency of mEPSCs from the control (n = 12 or 14), MEHP-100 (n = 12), and MEHP-300 (n = 12 or 13) groups, respectively. mEPSC were recorded with a standard external solution containing (in mM): 140 NaCl, 1.5 CaCl_2_, 5 KCl, 1 MgCl_2_, 10 HEPES, and 10 glucose (pH 7.2~7.4). Pipette electrodes were filled with internal solution containing (in mM): 140 potassium gluconate, 5 NaCl, 2 MgATP2, 1 CaCl_2_, 10 EGTA, and 10 HEPES (pH 7.2~7.4, 310 mOsm). A total of 1 μM tetrodotoxin (TTX) and 10 μM picrotoxin (PTX) were added into the standard external bath solution to block the Na^+^ currents and aminobutyric acidergic current, respectively. The data are expressed as the mean ± SEM. Each bar indicates the mean ± SEM from the indicated number of neurons. (** means *p* < 0.01, *** means *p* < 0.001).

Our present sAP, potassium current, and calcium data are consistent with our previous work [14,28], indicating that MEHP might have the ability to regulate synaptic plasticity. For functional synaptic plasticity of neurons, the frequency and amplitude of mEPSC are closely associated with the presynaptic neurotransmitter release and postsynaptic receptors’ numbers and strength, respectively [23]. Thus, we next recorded the mEPSC from CA3 neurons to investigate if MEHP regulated the synaptic plasticity. The typical traces of mEPSC from all experimental groups (control, MEHP-100, and MEHP-300) were shown in Figure 5A. At the concentrations of 100 and 300 μM, MEHP significantly reduced the frequencies of mEPSC from 6.33 ± 0.50 Hz to 3.55 ± 0.40 Hz and 2.61 ± 0.87 Hz, respectively (Figure 5B), but had no effects on the amplitude of mEPSC (Figure 5C) compared with control. The decrease of mEPSC frequency suggests that MEHP could inhibit the presynaptic function, for instance, of the properties of the calcium channel.

## 3. Discussion

Though DEHP and MEHP are reported to be toxic and hazardous to organisms’ health, they are still widely used in our daily lives. Due to the excellent stability of MEHP, epidemiological studies have shown that MEHP can be widely detected in human urine and blood, indicating the biosafety of DEHP/MEHP, an urgent public health concern. Unfavorable factors, such as aging and oxidative damage, will change the number and structure of synapses of the hippocampus, which is a major component of the brains of humans and other mammals, leading to neural dysfunctions, and the CA3 region of the hippocampus plays an important role in the formation of primary memory, and the process of nerve regeneration. Additionally, DEHP/MEHP has been demonstrated to have the ability to cross the blood–brain barrier [29,30], indicating the potential role of DEHP/MEHP on neural functions. But the effects of MEHP on neural function still remain unclear. In this work, we tested the electrophysiological properties of CA3 neurons from rat hippocampi with the treatments of MEHP to detect the effects of MEHP on the neural transmission. We found that MEHP modulates neuronal excitability and synaptic plasticity in the CA3 region neurons of rat hippocampi, consistent with the previous study, which revealed that acute DEHP exposure to rats reduced the axonal markers in CA3 distal stratum orient [31], implying that the hippocampus is hypersensitive to DEHP/MEHP.

In our study, we first detected the excitability changes of CA3 neurons after MEHP application (Figure 1). At a low concentration of MEHP (100 μM), only sAP amplitude was reduced, and at a higher concentration (300 μM), the potential threshold, amplitudes, peak time, and antipeak time were significantly decreased. sAP, as a brief of membrane potential, has fives phases, including rising, peak, falling, undershoot, and refractory period phases [32]. One of the most important factors, which will impact the formation of sAP, is the activity of the ion channel, and each type of ion channels has a specific junction in the processes of sAP. Thus, the changes of sAP with MEHP treatments suggested that MEHP might influence the neural excitability of CA3 neurons by mediating ion channels. In the following experiment, to detect how MEHP could mediate the ion channels activities, the sodium, potassium, and calcium channel recordings have been made. In the resting state, the resting membrane potential is determined by the movement of potassium ions [33]. Additionally, the sAP amplitude relies on the entry of sodium at the rising phase and exiting of potassium channel [34], and implying that MEHP might regulate the sodium activity. In our sAP data, the MEHP-induced depolarization and MEHP-induced amplitude reduction of sAP indicated that MEHP may have the ability to regulate sodium or potassium channels. Interestingly, our data showed that sodium current density and the potassium current density has been reduced by the higher concentration of MEHP (300 μM), but not lower concentration of MEHP (100 μM) treatment (Figure 2 and Figure 3), consistent with a decrease of sAP amplitude and depolarized CA3 neurons with MEHP treatment. For calcium channel recordings, our data showed that MEHP reduced the calcium current density after the MEHP treatment (Figure 4), consistent with the decrease of sAP antipeak time, which could be regulated by the influx of calcium ions [35]. Moreover, exposure to MEHP led to the sodium and calcium channels opening earlier and closing later, which means that channel gating is not the mechanism of MEHP that affects channel activity. Meanwhile, one of our previous studies found that DEHP could suppress the neuronal excitability and synaptic plasticity by inhibiting the voltage-gated potassium channel [14], indicating that MEHP might have the same mechanism as DEHP. Our other work found that DEHP could modulate the cholinergic mini-synaptic transmission of PNs from the Drosophila brain by inhibiting the calcium channel activities [13], suggesting that MEHP might have the ability to regulate the synaptic plasticity. Thus, in the following study, the mEHPCs of CA3 neurons with the treatment of MEHP-100 or MEHP-300 have been detected.

Synaptic plasticity mainly reflects the structural and functional changes on synaptic connections, which can improve the function of brain networks and enable the short- and long-term reshaping of neural transmission [36]. The frequency and amplitude of mEPSC present the properties of pre-synaptic receptors, involving calcium-regulated neurotransmitter release, and the properties of post-synaptic receptors, referring to the amount and intensity of receptors, respectively [37]. Our mEPSCs results showed that the frequency rather than the amplitude of mEPSC decreased significantly with the exposure to MEHP-100 and MEHP-300, implying the potential role of MEHP in the pre-synapsis properties of CA3 neural transmission. Combined with the changes of the calcium channel, MEHP might decrease the pre-synaptic properties by inhibiting the calcium channel.

At the stages of embryo and childhood, two important and sensitive periods of neurodevelopment, embryos and infants are more susceptible to harmful substances [4]. Because MEHP/ DEHP can access the fetus through the placental barrier, and, until 3 months of age, infants have immature glucuronidation pathways, MEHP is slow, so high excretion levels of MEHP in embryos and neonates can be detected [38]. Epidemiological studies have shown that the DEHP exposure levels of infants and children were much higher than those of adults and prenatal exposure to phthalates is closely related to the intellectual and mental development disorders in infants [39]. Although, the plasma levels of DEHP in the fractions obtained from the infants during transfusions (between 3.4 and 19.9 μg/mL) are slightly higher than the levels of MEHP in the same fractions (from 1.5 to 15.6 μg/mL), plasma concentrations of DEHP in these infants decreased at a faster rate than those of MEHP [22]. Moreover, it has been verified that DEHP may exert its toxic effects primarily via MEHP, which is tenfold more potent than its parent compound in toxicity assays in vitro [40]. Therefore, it is worthwhile to make an effort to explore the neurotoxicity effects of MEHP on human CNS and the mechanisms involved.

The present study demonstrated that exposure to MEHP does inhibit the neural excitability and synaptic plasticity of hippocampal neurons, implying that MEHP does play a distinct role in neural function. Although 100 μM MEHP had no significant effect on sodium and potassium channels, indicating that at the concentration of 50 μM (the maximal MEHP concentration in body fluids), MEHP will affect neither Na^+^ nor K^+^ current, the amplitude of sAP, the frequency of mEPSCs, and the Ca^2+^ currents were indeed significantly affected by 100 μM MEHP. Moreover, the effects of 50 μM MEHP on sAP, mEPSCs, and Ca^2+^ currents are unknown; that is to say that we need further study to explore whether MEHP could influence the excitability of hippocampal neurons at the concentration of the maximal MEHP in body fluids (50 μM), and to increase the physiological relevance of this study.

## 4. Materials and Methods

### 4.1. Animals

The Sprague-Dawley rats (2 to 3weeks old) were obtained from the Animal Laboratory Administrative Center of Chongqing Medical University, in accordance with the Institutional Animal Care and Use Committee. Animals in this study were maintained at 60% relative humidity under a 12-h light/dark cycle (7:00 a.m.~7:00 p.m.), at 25 °C with standard food and water. To avoid sex difference from influencing the results, the numbers of female and male mice rats used for the study were equal.

### 4.2. Drugs and Treatment

All chemicals, including MEHP, tetrodotoxin (TTX), picrotoxin (PTX), tetraethylammonium (TEA), 4-aminopyridine (4-AP), HEPES, EGTA, dimethyl sulfoxide (DMSO), and the chemical products used to prepare external and internal solutions were all purchased from Sigma–Aldrich Chemicals Co. (St. Louis, MO, USA). MEHP was dissolved in DMSO to give 200- and 600-mM stock solutions. The stock solutions of MEHP were further diluted in the external solution to the appropriate concentrations of 100 μM and 300 μM (shown as MEHP-100 and MEHP-300 in the text, respectively). The recordings of the control group were performed in the hippocampus neurons which were continuously perfused with the external solution with DMSO, and the recordings of MEHP-100 were subsequently tested on the same cell after washout, and the recordings of MEHP-300 were tested on another normal cell.

### 4.3. Brain Tissue Preparation

Hippocampal slices were obtained as described previously [14]. After being deeply anesthetized with 20% urethane, the brain was quickly removed and immersed into ice-cold oxygenated (95% O_2_/5% CO_2_, pH 7.4) artificial cerebrospinal fluid (ACSF) containing (in mM) 124 NaCl, 2.5 KCl, 2 CaCl_2_, 2 MgCl_2_, 1.25 NaH_2_PO_4_, 26 NaHCO_3_, and 10 glucose (300~310 mOsm). Then, transverse slices (350-μm-thick) were cut from hippocampus with a vibratome (Leica VT1000A; Wetzlar, Germany). The slice was incubated in ACSF for at least 1 h at room temperature to recover.

### 4.4. Whole-Cell Electrophysiological Recordings

Recordings were obtained from the CA3 hippocampal neurons as described by us before [15]. sAP, sodium current (Na^+^ current), potassium current (K^+^ current), total calcium current (Ca^2+^ current), and mini-excitatory postsynaptic currents (mEPSCs) have been recorded. Hippocampal slices were transferred to a submerged recording chamber (Warner Instruments, Hamden, CT, USA) and perfused continuously with ACSF at a rate of 3 mL/min. Pipette electrodes (tip resistance between 2~6 MΩ, Hilgenberg, Germany), were pulled by a Flaming–Brown electrode puller (P-97; Sutter Instruments,183 Novato, CA, USA). Giga-ohm seals were achieved prior to recording in on-cell configuration, followed by whole-cell recordings, by using an EPC 10 Amplifier-HEKA. Whole-cell recordings were performed with a standard external solution containing (in mM) 140 NaCl, 1.5 CaCl_2_, 5 KCl, 1 MgCl_2_, 10 HEPES, and 10 glucose (pH 7.2~7.4).

For sAP and mEPSC recordings, the pipette electrodes were filled with internal solution containing (in mM) 140 potassium gluconate, 5 NaCl, 2 MgATP2, 1 CaCl_2_, 10 EGTA, and 10 HEPES (pH 7.2~7.4, 310 mOsm). In current-clamp mode, sAP was recorded with the membrane potential holding at −70 mV. Only overshooting sAP more positive than 0 mV were selected, and the threshold, amplitude, frequency, peak time, antipeak amplitude, and antipeak time of sAP have been counted and analyzed. In voltage-clamp mode, mEPSC were recorded with the membrane potential holding at −70 mV. A total of 1 μM tetrodotoxin (TTX) and 10 μM picrotoxin (PTX) were added into the standard external bath solution to block the Na^+^ currents and aminobutyric acid-ergic current, respectively. Then mEPSC amplitudes (<20 pA) and mEPSC frequency within 2 min were analyzed.

For the Na^+^ current, pipettes were routinely filled with a solution containing (in mM) 140 CsCl, 10 TEA, 10 EGTA, 10 HEPES with pH adjusted to 7.2~7.4 using CsOH, and the extracellular recording solution consisted of the following (in mM): 140 NaCl, 1 MgCl_2_, 5.0 KCl, 3.0 CaCl_2_, 10 TEA, 1 4-AP, 0.2 CdCl_2_, 10 HEPES, 10 glucose with pH adjusted to 7.2~7.4 using NaOH (mOsm 290~300). For K^+^ current, pipettes were routinely filled with a solution containing (in mM) 140 KF, 10 EGTA, 10 HEPES with pH adjusted to 7.2~7.4 using KOH (mOsm 290~300), and an extracellular recording solution consisted of the following (in mM): 140 NaCl, 1.0 MgCl_2_, 5.0 KCl, 3 CaCl_2_, 0.001 TTX, 10 HEPES, 10 glucose with pH adjusted to 7.2~7.4 using NaOH (mOsm 300~310). For Ca^2+^ current, pipettes were routinely filled with an internal solution containing (in mM) 140 CsCl, 10 TEA, 2 Na2ATP, 10 EGTA, 10 HEPES with pH adjusted to 7.2~7.4 using CsOH (mOsm 290~300), and the extracellular recording solution consisted of the following (in mM): 140 NaCl, 1.0 MgCl_2_, 5.0 KCl, 3 CaCl_2_, 10 TEA, 1 4-AP, 0.001 TTX, 10 HEPES, 10 glucose with pH adjusted to 7.2~7.4 using NaOH (mOsm 300~310). Neurons were subjected to current-voltage (I-V) and activation/inactivation voltage protocols as follows: (a) I-V protocol: (i) Na^+^: from a −70 mV holding potential, cells were depolarized in 200-millisecond voltage steps from −100 to +80 mV (10-mV increments), (ii) K^+^: from a −70 mV holding potential, cells were depolarized in 200-millisecond voltage steps from −60 to +60 mV (10-mV increments), (iii) Ca^2+^: from a −70 mV holding potential, cells were depolarized in 150-millisecond voltage steps from −70 to +60 mV (10-mV increments), which allowed acquisition of current density values such that we analyzed activation of sodium, potassium, and calcium channels as a function of current vs. voltage and infer peak current density (normalized to cell capacitance (in picofarads, pF)); (b) inactivation protocol: Na^+^: from a holding potential of −70 mV, cells were subjected to hyperpolarizing/repolarizing pulses for 1 second between −120 to 10 mV (10-mV increments), Ca^2+^: from a holding potential of −70 mV, cells were subjected to hyperpolarizing/repolarizing pulses for 1 second between −100 to 30 mV (10-mV increments). Analysis was performed by using Fitmaster software (HEKA) and Origin 9.0 software (OriginLab).

### 4.5. Statistics

Values are presented as mean ± SEM. *p* < 0.05 was considered to be significant. All data was first tested for a Gaussian distribution using a D’Agostino–Pearson test (Prism 8 Software, Graphpad, San Diego, CA, USA). The statistical significance of differences between means was determined by parametric ANOVA followed by Tukey’s post hoc or non-parametric Kruskal–Wallis test followed by Dunn’s post-hoc test depending on whether datasets achieved normality. All data were plotted in Prism 8.

## 5. Conclusions

The present study demonstrated that MEHP does inhibit the neural excitability and synaptic plasticity of hippocampal neurons, implying that MEHP does play a distinct role in neural function. However, the specific molecular mechanisms of MEHP on the central nervous system are still unknown, and further exploration is needed.

## Data Availability

Not applicable.

## Sample Availability

Sample of the MEHP is available from Sigma (America). Product No 4376-20-9.
